# Characterization of butyrate-metabolism in colorectal cancer to guide clinical treatment

**DOI:** 10.1038/s41598-023-32457-z

**Published:** 2023-03-29

**Authors:** Qinghua Luo, Ping Zhou, Shuangqing Chang, Zhifang Huang, Xuebo Zeng

**Affiliations:** 1 Department of Anorectal Surgery, Jiangmen Wuyi Hospital of Traditional Chinese Medicine, Jiangmen, China; 2 Department of Anorectal Surgery, Jiangxi Hospital of Integrated Traditional Chinese and Western Medicine, Nanchang, China; 3 Department of Brain Diseases, Shenzhen Pingle Orthopaedic Hospital, Shenzhen, China

**Keywords:** Cancer models, Oncology

## Abstract

Colorectal cancer (CRC) is the third most prevalent one in the world among the most common malignant tumors. Numerous studies have shown that butyrate has demonstrated promise as an antitumor agent in a variety of human cancer types. However, butyrate remains understudied in CRC tumorigenesis and progression. In this study, we explored therapeutic strategies to treat CRC by examining the role of butyrate metabolism. First, from the Molecular Signature Database (MSigDB), we identified 348 butyrate metabolism-related genes (BMRGs). Next, we downloaded 473 CRC and 41 standard colorectal tissue samples from The Cancer Genome Atlas (TCGA) database and the transcriptome data of GSE39582 dataset from Gene Expression Omnibus (GEO) database. Then we evaluated the expression patterns of butyrate metabolism-related genes with difference analysis in CRC. Through univariate Cox regression and least absolute shrinkage and selection operator (LASSO) analysis, a prognostic model was constructed based on differentially expressed BMRGs. In addition, we discovered an independent prognostic marker for CRC patients. According to the expression levels and coefficients of identified BMRGs, the risk scores of all CRC samples were calculated. Utilizing differentially expressed genes in the high- and low-risk groups, we also constructed a Protein–Protein Interaction (PPI) network to visualize the interactions between proteins. Through the results of PPI network, we screened out differentially expressed target butyrate metabolism-related genes from ten hub genes. Finally, we performed clinical correlation analysis, immune cell infiltration analysis, and mutation analysis for these target genes. One hundred and seventy three differentially expressed butyrate metabolism-related genes were screened out in all the CRC samples. The prognostic model was established with univariate Cox regression and LASSO regression analysis. CRC patients’ overall survival was significantly lower in the high-risk group than in the low-risk group for both training and validation set. Among the ten hub genes identified from the PPI network, four target butyrate metabolism-related genes were identified containing FN1, SERPINE1, THBS2, and COMP, which might provide novel markers or targets for treating CRC patients. Eighteen butyrate metabolism-related genes were used to develop a risk prognostic model that could be helpful for doctors to predict CRC patients’ survival rate. Using this model, it is beneficial to forecast the response of CRC patients to immunotherapy and chemotherapy, thus making it easier to custom tailor cancer chemotherapy and immunotherapy to the individual patient.

## Introduction

In 2020, a total of 1,148,515 new colon cancer patients were diagnosed around the world, or 6% of new cancer cases; 576,858 new colon cancer deaths were reported, representing 5.8% of all cancer deaths all over the world according to the World Health Organization report^1^. With the rapid advancement of science and technology, medical treatment methods are changing rapidly. In addition to the two conventional faecal occult blood testing and colonoscopy, faecal genetic testing is now employed in pre-colorectal-cancer screening^2^. In terms of common colorectal surgical procedures, Laparoscopic assisted surgery and da Vinci robot-assisted surgery are replacing orthodox open surgery^3^. In terms of postoperative chemotherapy regimens, patients with metastatic or advanced disease are often treated with biologic agents that reinforce cytotoxic therapy including bevacizumab and cetuximab, in addition to FOLFOX6 and CapeOX regimens^4^. Nevertheless, the prognosis of each CRC patient is highly heterogeneous, as their genetic characteristics and different risk factors can lead to inconsistent disease progression and varying therapy outcomes, particularly in cases of recurrent postoperative CRC, where surgery and chemotherapy are not beneficial^5,6^. Besides, a poor prognosis is associated with CRC in its advanced stages^7^. More importantly, as a result of the rapid growth in developing countries, Arnold et al. have forecast that there will be 2.5 million cases of CRC in 2035^8^. Therefore, it is indispensable for us to hunt for a novel method to better predict the prognosis of CRC patients.

A short-chain fatty acid (SCFA), butyrate is formed by bacteria in the colon during fermentation of fibre, and is used by colonocytes for energy^9^. By promoting colonic motility, butyrate accelerates blood flow to the colon and reduces colonic anastomosis healing time indirectly^10^. The use of butyrate reduces intestinal permeability, improving intestinal mucosal barrier function and improving immune function, which is very beneficial for ulcerative colitis and other conditions that affect the intestinal wall^11^. Butyrate plays an essential role in intestinal cancer by providing nutrition to healthy mucosa, promoting its proliferation, and maintaining good intestinal barrier condition by accelerating mucus production. In metabolomic and proteomic studies, researchers found that butyrate inhibited CRC cell proliferation by directly targeting pyruvate kinase M2 and subsequently reprogramming metabolism^12^. Combining butyrate with the G protein-coupled receptor GPR109A can also suppress tumors in the colon^13^. Metabolic pathway analyses in CRC can provide us with a better understanding of the molecular mechanisms involved and provide us with new and more effective therapeutic approaches^14^. Currently, there are no systematic studies on butyrate metabolism-related genes in CRC.

In the present study, we constructed a prognostic model based on butyrate metabolism-related genes using univariate Cox regression and LASSO regression analysis, which was validated by the GEO-CRC cohort. CRC patients’ overall survival outcomes were comprehensively predicted and analyzed using this prognostic model. We also analyzed the differences between high-risk and low-risk CRC patients regarding immune cell infiltration, gene mutation, chemotherapeutic drug sensitivity, and immunotherapy efficacy. Next, we constructed a PPI network to identify ten hub genes utilizing differentially expressed genes in the high- and low-risk groups. Four target butyrate metabolism-related genes were identified from 10 hub genes, which might provide novel markers or targets for treating CRC patients. Further, we performed an in-depth comparison of these four genes in terms of survival prognosis, clinical characteristics and immune cells. The prognostic model has the potential to guide prognostic prediction and clinical medication of CRC patients.

## Materials and methods

### Data collection and collation

First, we identified a total of 348 butyrate metabolism-related genes from the Molecular Signature Database (MSigDB, https://www.gsea-msigdb.org/gsea/msigdb/index.jsp) by using “butyric acid” as a search term^15^. Subsequently, through the TCGA database (https://portal.gdc.cancer.gov/), we performed the download of raw RNA sequencing (RNA-seq) data profiles and obtained relevant data (survival status, follow-up time, sex, age, pathological stage, and TNM stage) for 473 CRC and 41 standard colorectal tissue samples. Through the GEO database (https://www.ncbi.nlm.nih.gov/geo/), we downloaded the transcriptional data and corresponding clinical data of CRC samples (gene ID: GSE39582). The gene IDs of the samples were converted to the related gene symbols using human gene annotation files.

### Identification of differentially expressed butyrate metabolism-related genes

We set |Log2fold change (FC)|> 0.585 and false discovery rate (FDR) < 0.05 as a threshold to screen out differentially expressed butyrate metabolism-related genes through performing the “limma” package in R software.

### Functional enrichment analysis of differentially expressed butyrate metabolism-related genes

To better investigate the biological characteristics and functional cellular pathways of differentially expressed butyrate metabolism-related genes, we performed Gene Ontology (GO) and Kyoto Encyclopedia of Genes and Genomes (KEGG) analysis with p-value < 0.05 for statistical significance^16,17^. Finally, we used two R packages “ggplot 2” and “goplot” to visualize the enrichment analysis results. GO enrichment analysis is divided into three major functional aspects, called GO types, including biological process (BP), cellular component (CC), molecular function (MF).

### Identification of prognostic genes

We used TCGA-CRC cohort samples as a training set. In order to identify the prognostic differentially expressed genes associated with butyrate metabolism, we set a p-value lower than 0.05. The “survival” package was used to identify the association between gene expression levels and patients’ overall survival using univariate Cox regression analysis on differentially expressed butyrate metabolism-related genes of the training set. The number of genes was further reduced and gene collinearity was eliminated using LASSO Cox regression. Moreover, we analyzed the intrinsic relationship between mutation frequency and mutated genes in the training set using the “maftools” R package.

### Principal component analysis

We used the “limma” package in R software for principal component analysis to better differentiate CRC patients between high and low-risk groups. We used the “ggplot2” package to obtain two-dimensional principal component analysis plots of the two principal components based on the expression profiles of genes related to butyrate metabolism and the gene expression profiles of the predictive risk score model, respectively.

### Construction and validation of a prognostic model

The risk scores of all samples were calculated using the following equation based on the results of LASSO Cox regression: risk score = Coef_1_ × ExpGene_1_ + Coef_2_ × ExpGene_2_ + Coef_3_ × ExpGene_3_ + … + Coef_i_ × ExpGene_i_.

“Coef” corresponds to the non-zero regression coefficient obtained by LASSO Cox regression analysis, and “ExpGene” corresponds to the expression value of the gene in the prognostic risk score model. All the CRC patients were divided into low-risk and high-risk groups based on the median risk score. Kaplan–Meier survival plots were calculated using the R package “survival”. In order to evaluate the prognostic ability of the model, a receiver operating characteristic (ROC) curve was generated using the timeROC package. We validated the results using samples from the GEO database simultaneously. The same formula used in the training set was applied to patient risk scores from the GEO cohort. Next, we investigated whether CRC risk score was an independent predicator of overall survival using univariate and multivariate independent prognostic analysis. Statistical significance was determined by a p-value less than 0.05.

### Construction and evaluation of the nomogram of CRC patients

To build a predictive model based on independent clinical parameters that can be used intuitively to study the overall survival of individual CRC patients, we used the “nomogram” package in R for this process. The relationship between risk score and clinical information was explored using the “limma” package, which included gender, age, pathological stage, and TNM stage. Calibration plots and ROC curves were used to assess the predictive performance of this model. To determine whether nomogram could be a reliable independent prognostic indicator for CRC patients, univariate and multivariate independent analyses were performed. P-value of < 0.05 was used to show statistical significance.

### Characteristics of patients in high- and low-risk groups

Using the “ggpubr” package, we obtained a correlation between patients’ risk score and tumor mutation frequency. To estimate the relationship between immune cell infiltration and risk score, we downloaded the immune cell infiltration files from Timer2.0 (http://timer.cistrome.org/), and then used the “limma” and “pheatmap” R packages for differential analysis. The “GSVA” and “GSEABase” packages were used to analyze the differences in immune-related functions between the high-risk and low-risk groups. To predict the effect of immunotherapy in the high and low-risk groups, we used the TIDE online database (http://tide.dfci.harvard.edu/). P-values of less than 0.05 indicated a statistically significant difference.

### Identification of potential drugs for CRC patients

The “PRRophetic” R package was used to predict the semi-inhibitory concentrations of drugs in the high-risk and low-risk groups and identify drugs with differential efficacy.

### Protein–protein interaction network and target gene characteristics

Using the STRING online database (https://cn.string-db.org/), the interaction network of these differentially expressed genes between high and low-risk groups were mapped (medium confidence > 0.40). Using the cubHubba plugin of Cytoscape software (version 3.9.1), we screened out the top 10 network core genes. Using the GEPIA (http://gepia.cancer-pku.cn) online database, differentially expressed target genes in tumor tissues and normal tissues were filtered from the top 10 core genes by setting threshold log2FC > 1 and p-value < 0.05. To analyze the infiltration of the target genes in 22 tumour-infiltrating lymphocytes in the microenvironment of high-risk and low-risk CRC patients, the CIBERSORTx online database (https://cibersortx.stanford.edu/index.php) was used, and then “reshape2 “reshape2” and “ggpubr” R packages were used to map out the differential results. The “limma” and “ggpubr” R packages were used to analyze the relationship between target gene expression and clinical characteristics (age, sex, grade, TNM stage) in CRC patients. Finally, we performed an in-depth study of the correlation between target genes using the GEPIA online database with Spearman test.

## Results

### Enrichment analysis of differentially expressed butyrate metabolism-related genes

**Figure 1:**
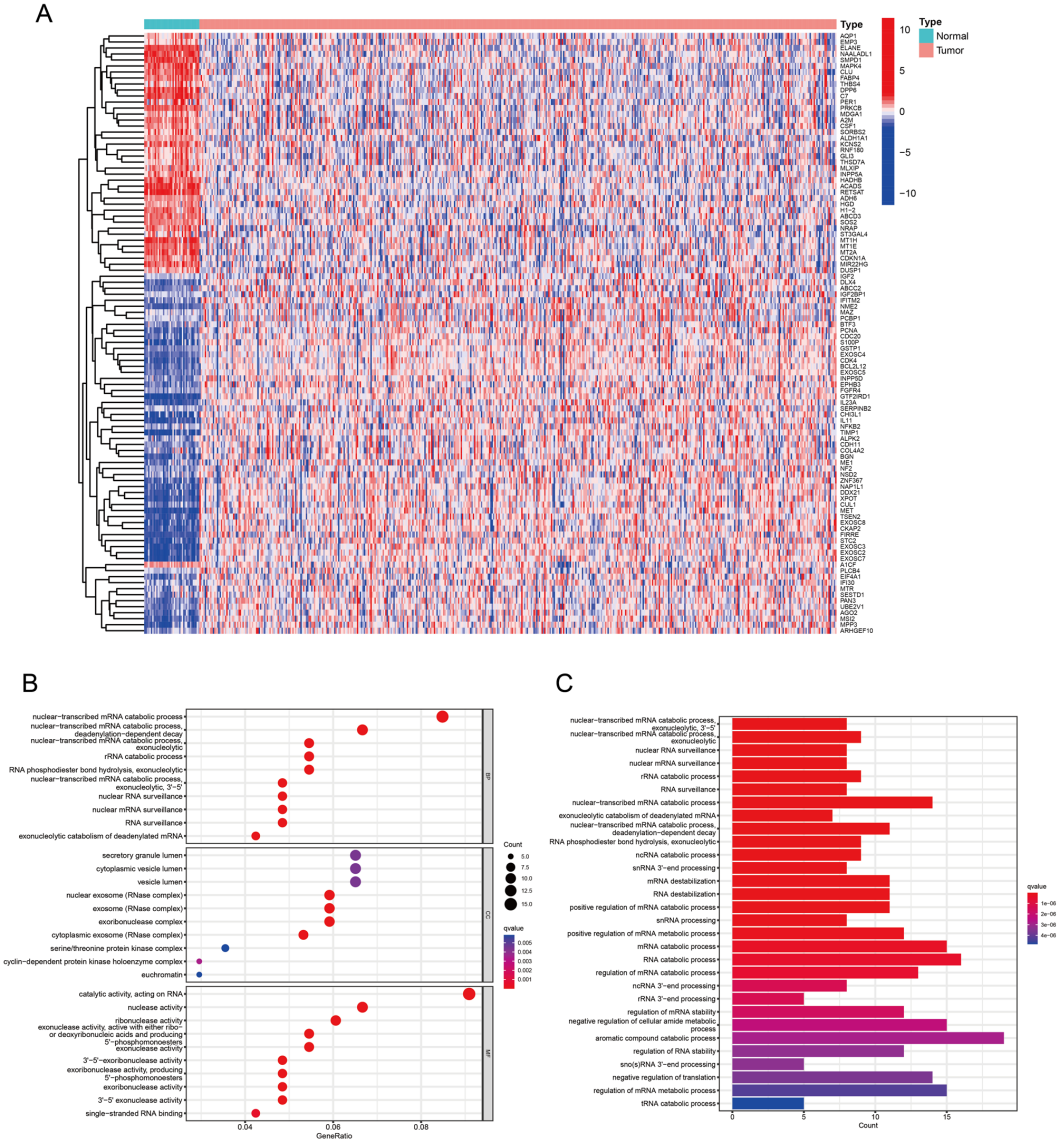
Analysis of differentially expressed butyrate metabolism-related genes. (**A**) Heatmap of differentially expressed butyrate metabolism-related genes. (**B**) GO analysis of differentially expressed butyrate metabolism-related genes. (**C**) KEGG analysis of differentially expressed butyrate metabolism-related differential genes.

Gene expression levels were compared between normal colorectal and cancerous tissue samples related to butyrate metabolism. We screened out a total of 173 differentially expressed butyrate metabolism-related genes between CRC and normal colorectal tissues, including 132 upregulated genes and 41 down-regulated genes. The distribution of these differentially expressed butyrate metabolism-related genes in normal and tumor samples were visualized by the heatmap (Fig. 1A). According to the visualization chart of GO enrichment analysis, we can see that in terms of BP, the differentially expressed butyrate metabolism-related genes were mostly gathered in nuclear-transcribed mRNA catabolic process, nuclear-transcribed mRNA catabolic process, deadenylation- dependent decay. In terms of CC, the differentially expressed butyrate metabolism-related genes were mostly gathered in nuclear exosome (RNase complex), cytoplasmic exosome (RNase complex), and exosome (RNase complex). In terms of MF, the differentially expressed butyrate metabolism-related genes were mostly gathered in 3' − 5' − exoribonuclease activity, exoribonuclease activity, producing 5' − phosphomonoesters, and exoribonuclease activity (Fig. 1B). According to the visualization of KEGG enrichment analysis, differentially expressed butyrate metabolism-related genes were mostly gathered in nuclear − transcribed mRNA catabolic process exonucleolytic 3' − 5', nuclear − transcribed mRNA catabolic process exonucleolytic, nuclear RNA surveillance (Fig. 1C). From the above results, we found that these differentially expressed butyrate metabolism-related genes in CRC are closely related to RNA metabolism.

### Construction of a prognostic model in the train set

**Figure 2:**
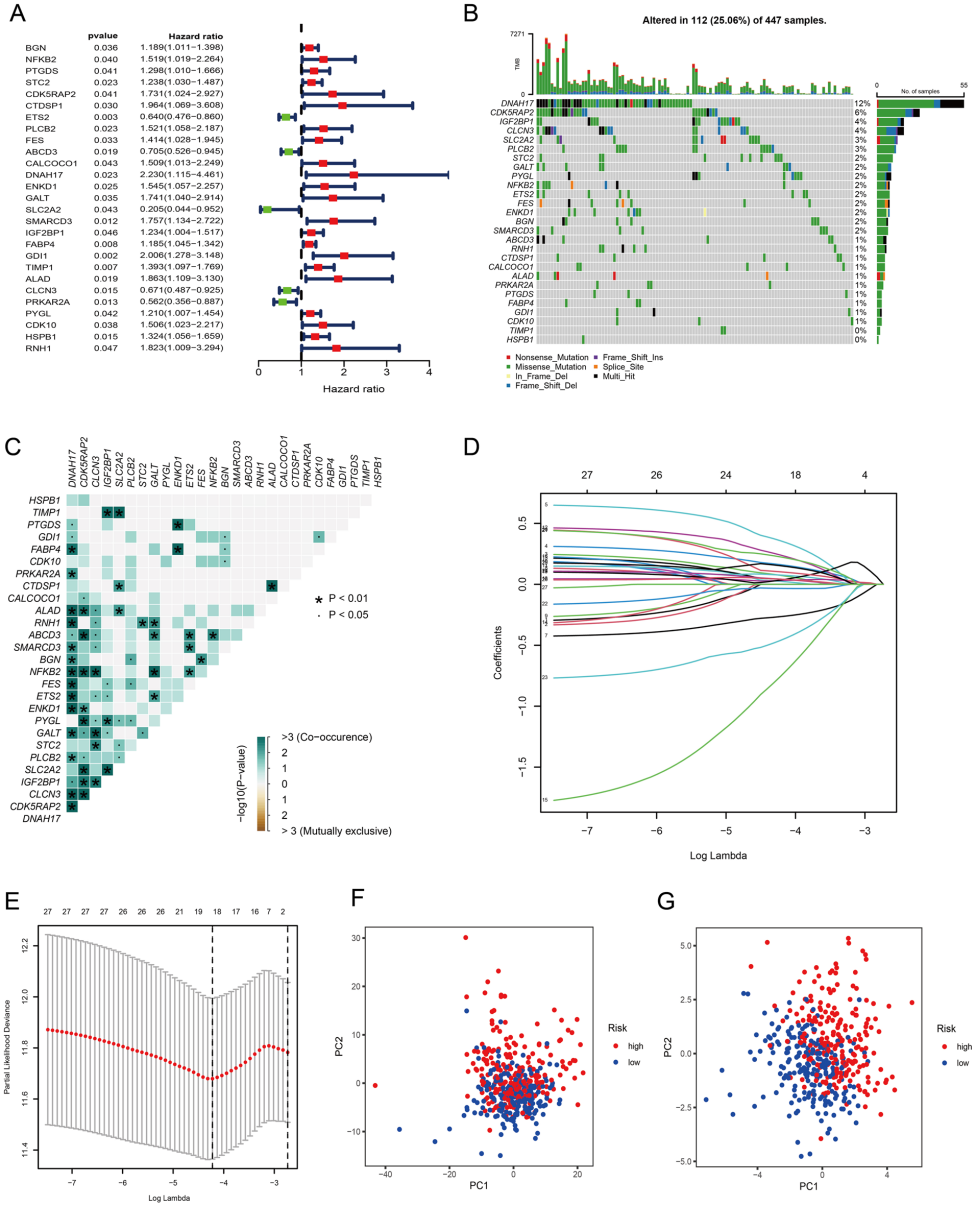
Identification of prognostic genes. (**A**) Univariate Cox regression analysis of butyrate metabolism-related genes. When the hazard ratio of a gene is > 1, it indicates that the gene is a risk factor for the corresponding tumor, and vice versa. (**B**) Gene mutations in CRC patients. (**C**) Correlation of mutations in 27 butyrate metabolism-related genes. Brown color indicates negative correlation, and blue color indicates positive correlation. (**D**) LASSO coefficient spectrum of 27 butyrate metabolism-related genes. (**E**) Cross-validation of adjustment parameter selection in a proportional hazards model. (**F**) PCA based on all butyrate metabolism-related genes. (**G**) PCA based on butyrate metabolism-related model genes. The red group represents high-risk patients, and the blue group represents low-risk patients.

Our training set was made up of TCGA-CRC cohort samples. We selected 27 butyrate metabolism-associated genes associated with prognosis from the 173 differentially expressed butyrate metabolism-associated genes with p value < 0.05 using univariate Cox regression analysis (Fig. 2A). The somatic mutation profiles of CRC patients were drawn for 27 genes linked to butyrate metabolism. There were 112 mutations in butyrate metabolism-related genes in 447 CRC samples, resulting in a 25.06 percent frequency (Fig. 2B). The more apparent mutations could be observed in DNAH17, CDK5RAP2, IGF2BP1, CLCN3, SLC2A2, and PLCB2, while the corresponding mutation frequencies were 12%, 6%, 4%, 4%, 3% and 3%, respectively. The visualization of co-mutations revealed a mutational positive relationship between DNAH17 and most of the other genes (Fig. 2C). Then, eighteen genes (PTGDS, STC2, CDK5RAP2, ETS2, CALCOCO1, DNAH17, ENKD1, SLC2A2, IGF2BP1, FABP4, GDI1, TIMP1, ALAD, CLCN3, PRKAR2A, PYGL, CDK10, and HSPB1) were screened out to construct a prognostic model using LASSO Cox regression analysis (Fig. 2D,E). The prognostic model was built according to the following risk score formula: Riskscore = (0.0744 * PTGDS) + (0.1617 * STC2) + (0.3586 * CDK5RAP2) + (− 0.2655 * ETS2) + (0.0244 * CALCOCO1) + (0.2413 * DNAH17) + (0.0420 * ENKD1) + (− 0.7597 * SLC2A2) + (0.0762 * IGF2BP1) + (0.0308 * FABP4) + (0.0344 * GDI1) + (0.1029 * TIMP1) + (0.2256 * ALAD) + (− 0.0333 * CLCN3) + (− 0.4618 * PRKAR2A) + (0.0428 * PYGL) + (0.1531 * CDK10) + (0.0426 * HSPB1). The CRC samples in TCGA were distinguished between the low-risk and high-risk groups by this risk model (Fig. 2F,G).

### The relationship between risk score and clinical features

**Figure 3:**
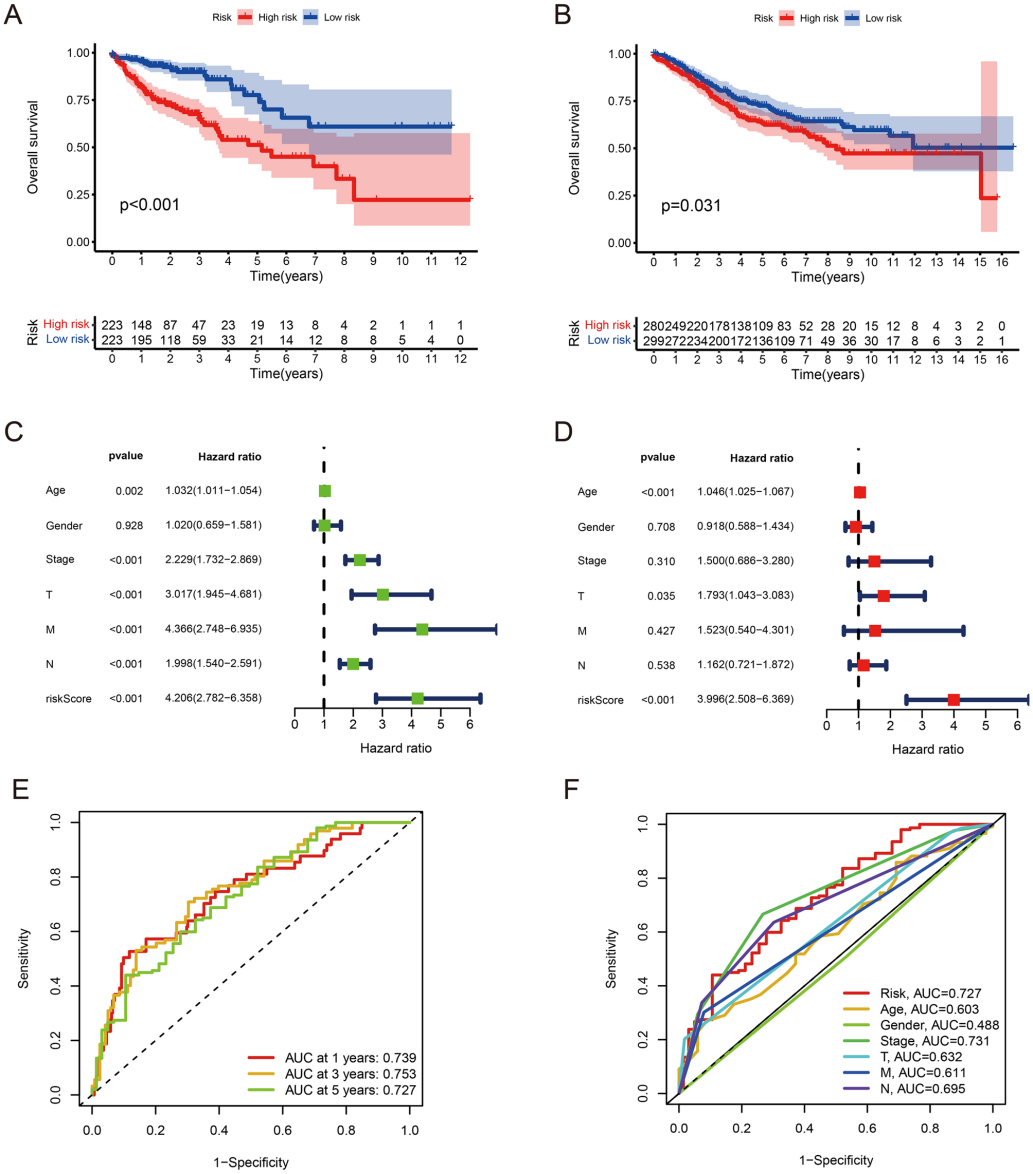
Predictive value of butyrate metabolism risk score in CRC survival. (**A**) Overall survival by butyrate metabolism risk score in the TCGA-CRC cohort. (**B**) Overall survival by butyrate metabolism risk score in the GEO-CRC cohort. (**C**) Results of univariate independent prognostic analysis in the TCGA-CRC cohort. (**D**) Results of multivariate independent prognostic analysis in the TCGA-CRC cohort. (**E**) AUC values at 1, 3, and 5 years in the TCGA-CRC cohort. (**F**) ROC curves of risk scores and clinical characteristics in the TCGA-CRC cohort.

**Figure 4:**
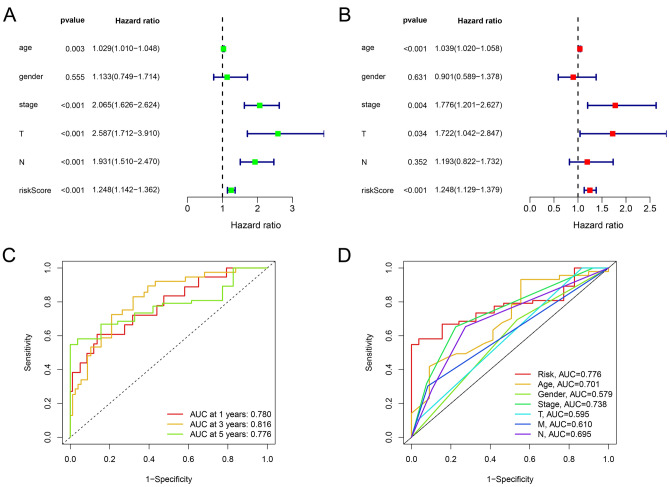
Validation of the prognostic model in GSE39582. (**A**) Univariate independent prognostic analysis in the GSE39582 cohort. (**B**) Multivariate independent prognostic analysis in the GSE39582 cohort. (**C**) AUC values at 1, 3 and 5 years in the GSE39582 cohort. (**D**) ROC curves of risk scores and clinical characteristics in the GSE39582 cohort.

In the constructed risk model, we divided CRC patients into two groups on the basis of their median risk scores in the training set and test set. The clinical prognosis of the low-risk group was found to be better than that of the high-risk group in both the training and test groups (p-value < 0.05) (Fig. 3A,B). Univariate and multivariate independent prognostic analysis were performed on the training set to investigate whether risk score could be an independent factor to predict the overall survival of CRC patients. The results indicated that age, T stage, and risk score could be independent factors to predict the overall survival of CRC patients (Fig. 3C,D). In assessing the reliability of the risk score of the prognostic model, we plotted receiver operating characteristic (ROC) curves and the area under the curves (AUCs) of CRC patients were 0.739, 0.753 and 0.727 for 1-, 3- and 5-year, respectively (Fig. 3E). The AUC value of the risk score was only slightly lower than the AUC value of the stage, which could indicate that the accuracy of the risk model is excellent (Fig. 3F). Furthermore, the prognostic model was validated in GSE39582 dataset. The result demonstrated that risk score and tumor stage were strongly correlated with overall survival of CRC patients based on univariate and multivariate Cox analyses (Fig. 4A,B). AUC values of CRC patients were 0.780, 0.816, 0.776 for 1-, 3- and 5-year, respectively (Fig. 4C). Meanwhile, Fig. 4D indicating that our prognostic model could have a higher prognostic value than traditional models. Considering the above results, we are able to conclude that risk score is a reliable independent indicator for predicting the overall survival rate of patients with CRC.

### Construction and evaluation of nomogram

**Figure 5:**
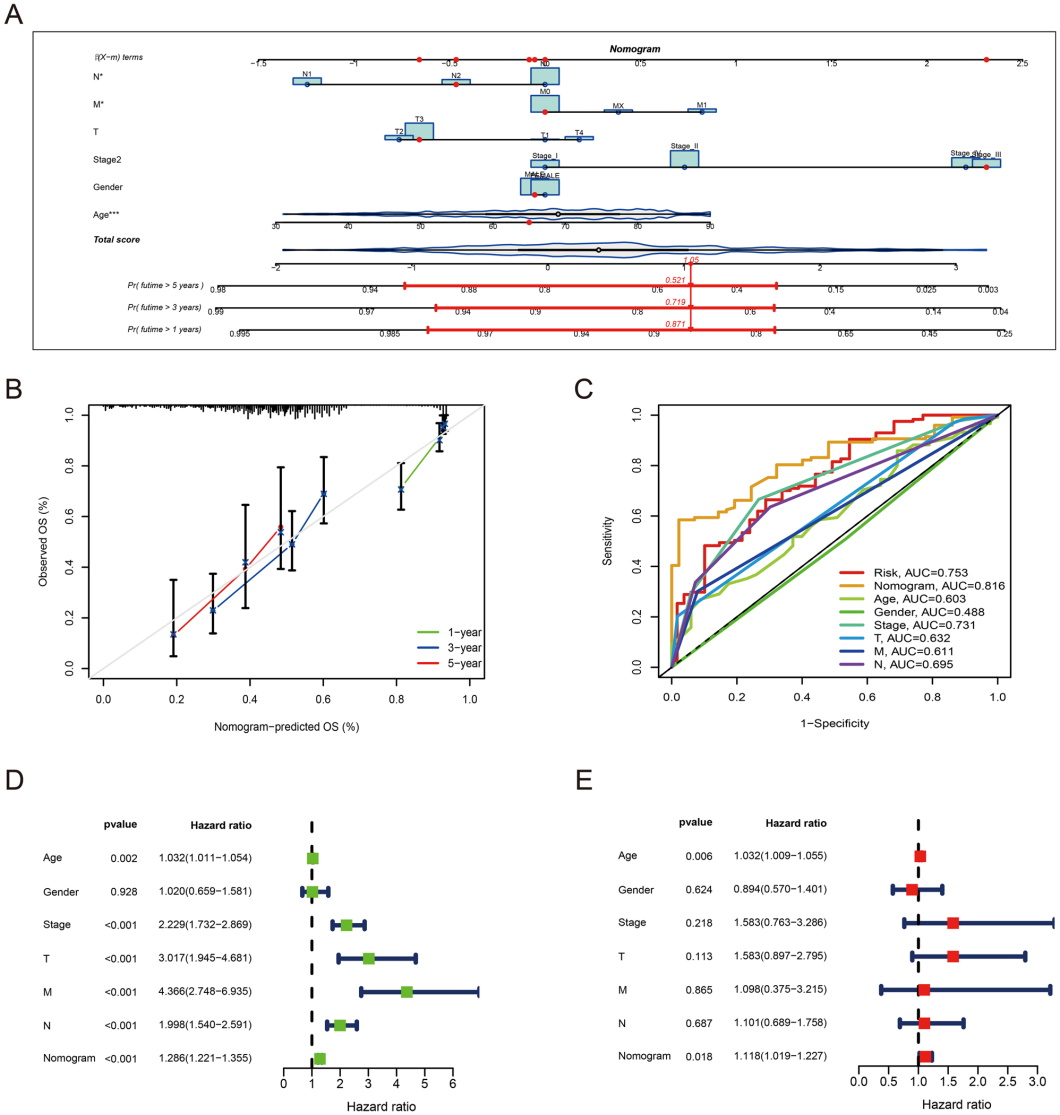
Construction of nomogram. (**A**) Nomogram to predict 1-, 3-, and 5-year overall survival in the TCGA-CRC cohort. (**B**) Calibration curves to assess the accuracy of a nomogram. (**C**) ROC curves of nomogram and clinical characteristics in the TCGA-CRC cohort. (**D**) Univariate independent prognostic analysis to determine whether the nomogram can be used as an independent prognostic factor. (**E**) Multivariate independent prognostic analysis to determine whether the nomogram can be used as an independent prognostic factor.

A nomogram including risk score, grade, TNM stage, age, and gender can predict the 1-year, 3-year, and 5-year survival rates of patients with CRC **(**Fig. 5A). A calibration curve at 1 year, 3 years, and 5 years demonstrated that the nomogram accurately predicted the overall survival of CRC patients (Fig. 5B). It was evident from the ROC curve that AUC value of nomogram (AUC = 0.816) was superior to any single indicator (Fig. 5C). The results of univariate and multivariate independent prognostic analysis suggest our nomogram could be an independent factor to predict the overall survival of CRC patients (p-value < 0.05) (Fig. 5D,E). In conclusion, the predictive accuracy of the presently constructed prognostic model is confirmed by the above aspects.

### Immune-related characteristics between the low- and high-risk groups

**Figure 6:**
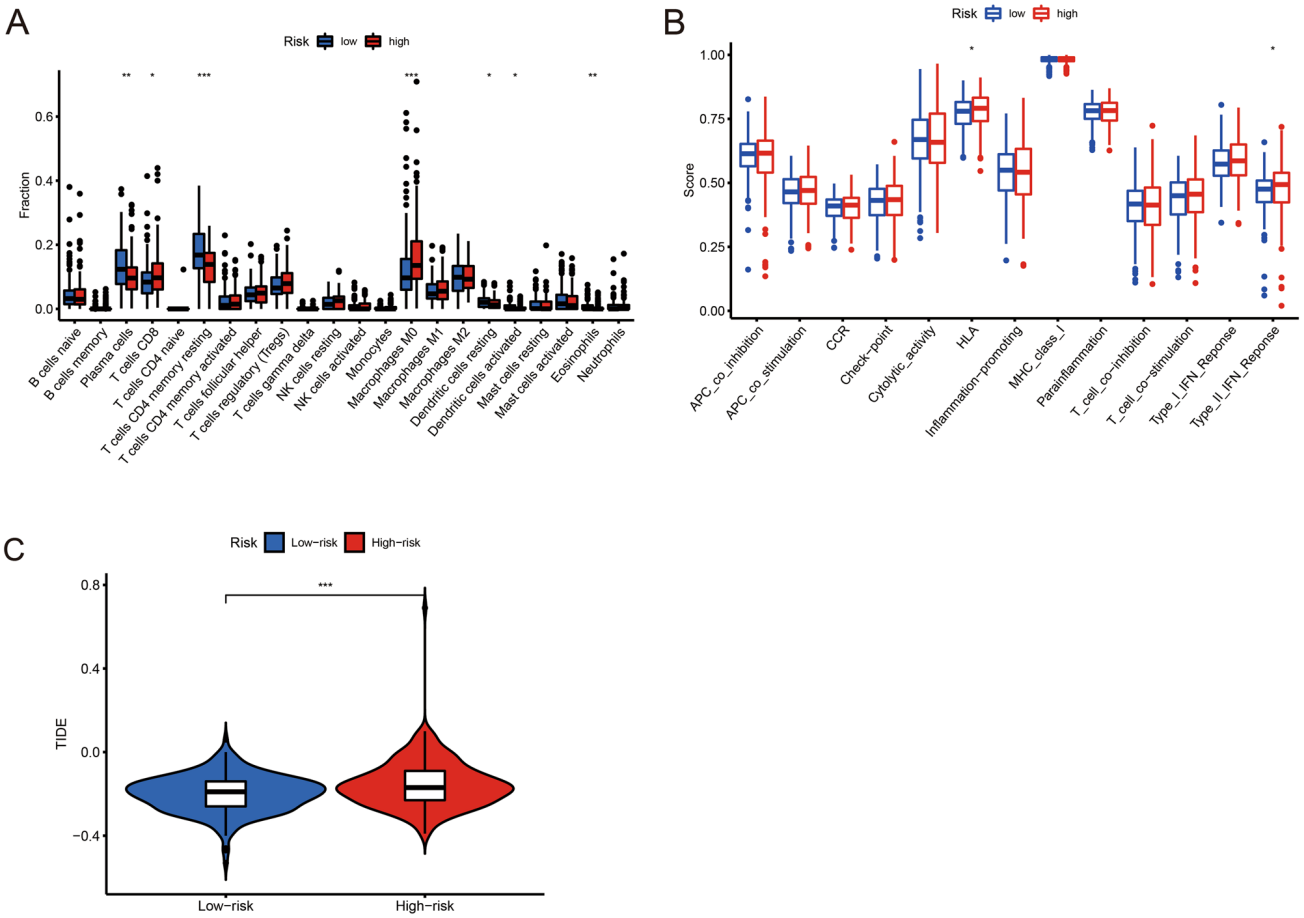
Immune-related analysis between high-risk and low-risk groups. (**A**) The immune infiltration of immune cell types in high-risk and low-risk patients in the TCGA-CRC cohort. (**B**) Analysis of immune functions in high-risk and low-risk patients in the TCGA-CRC cohort. (**C**) High-risk and low-risk CRC patients with TIDE scores in the TCGA-CRC cohort. *p < 0.05, **p < 0.01, ***p < 0.001.

The immune cells upregulated in the low-risk group were Plasma cells, CD4 memory resting T cells, resting Dendritic cells, activated Dendritic cells, and Eosinophils (p-value < 0.05), while upregulated immune cells in the high-risk group were CD8 T cells, Macrophages M0 (p-value < 0.05) (Fig. 6A). Immune function analysis showed that the high-risk group was active in HLA, Type II IFN Reponse immune-related functions (p-value < 0.05) (Fig. 6B). In the graph of the Tumor Immune Dysfunction and Exclusion (TIDE) score, the score in the high-risk group was higher than in the low-risk group (Fig. 6C), which could indicate that the high-risk group was more prone to immune escape, and their immunotherapy was less effective.

### Potential drugs for CRC patients

**Figure 7:**
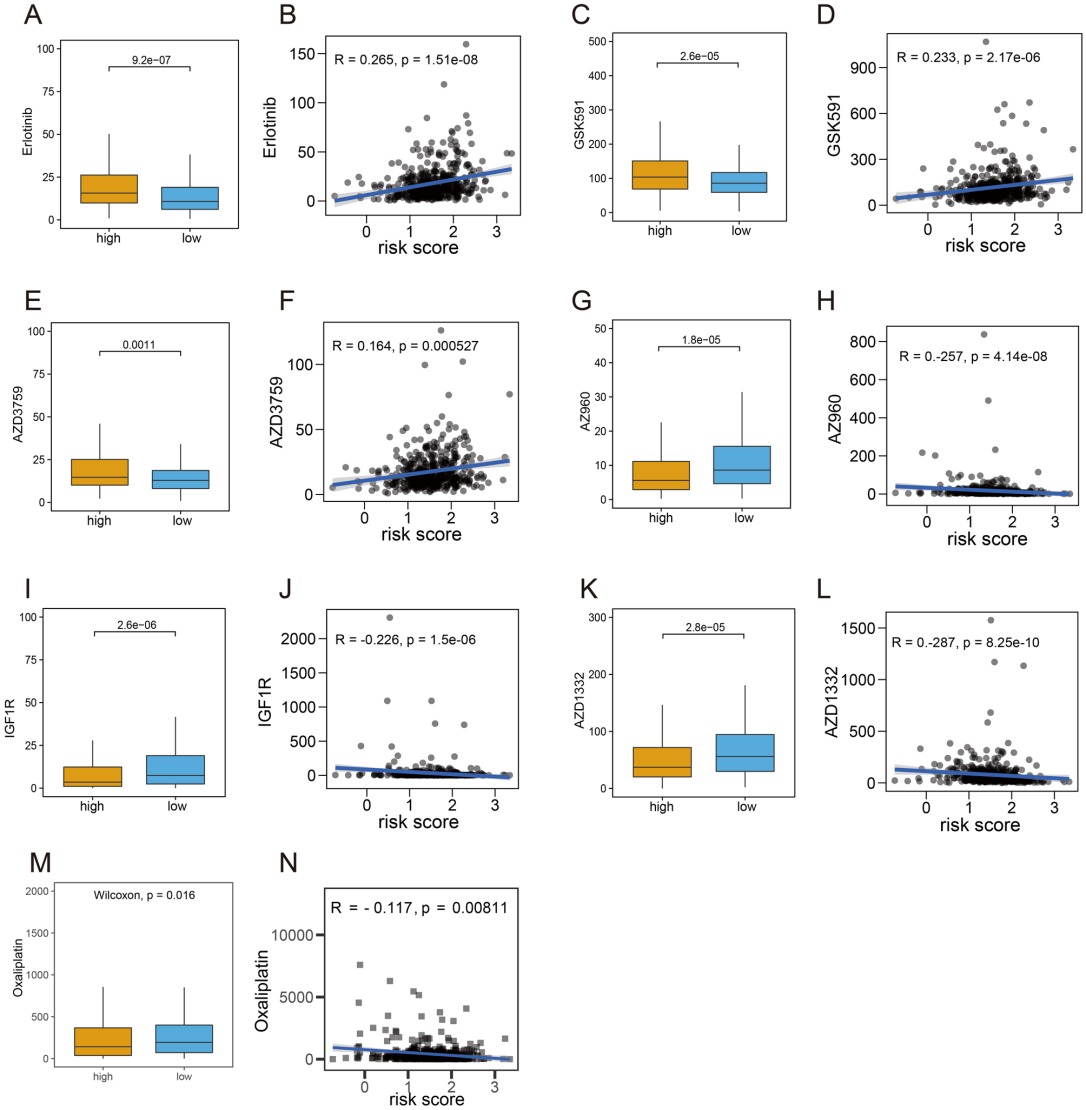
Identification of potential drugs. (**A,C,E,G,I,K,M**) Drug sensitivity analysis between high- and low-risk groups. (**B,D,F,H,J,L,N**) The relationship between butyrate metabolism risk scores and drugs.

In the treatment of patients with CRC, potential drugs may be special targets. In this study, we obtained a total of 58 drugs with statistical difference including 19 drugs more sensitive to the low-risk group and 39 drugs more sensitive to the low-risk group (Supplementary Table 1). The top 3 drugs more sensitive to the low-risk group were Erlotinib, GSK591, and AZD3759 (Fig. 7A,C,E), while the top 3 drugs more sensitive to the high-risk group were IGF1R, AZ960, and AZD1332 (Fig. 7G,I,K). Interestingly, the relationship between risk score and 3 drugs more sensitive to the low-risk group were positively correlated (Fig. 7B,D,F), while the relationship between risk score and 3 drugs more sensitive to the high-risk group were negatively correlated (Fig. 7H,J,L). Oxaliplatin, a commonly used chemotherapeutic agent in CRC patients, was more sensitive in the high-risk group (p-value < 0.05) (Fig. 7M), and the relationship between risk score and its chemotherapeutic drug sensitivity was negatively correlated (Fig. 7N**)**. In conclusion, these drugs can be beneficial in treating patients with CRC by providing new targets.

### PPI network of differentially expressed genes in low- and high-risk groups

**Figure 8:**
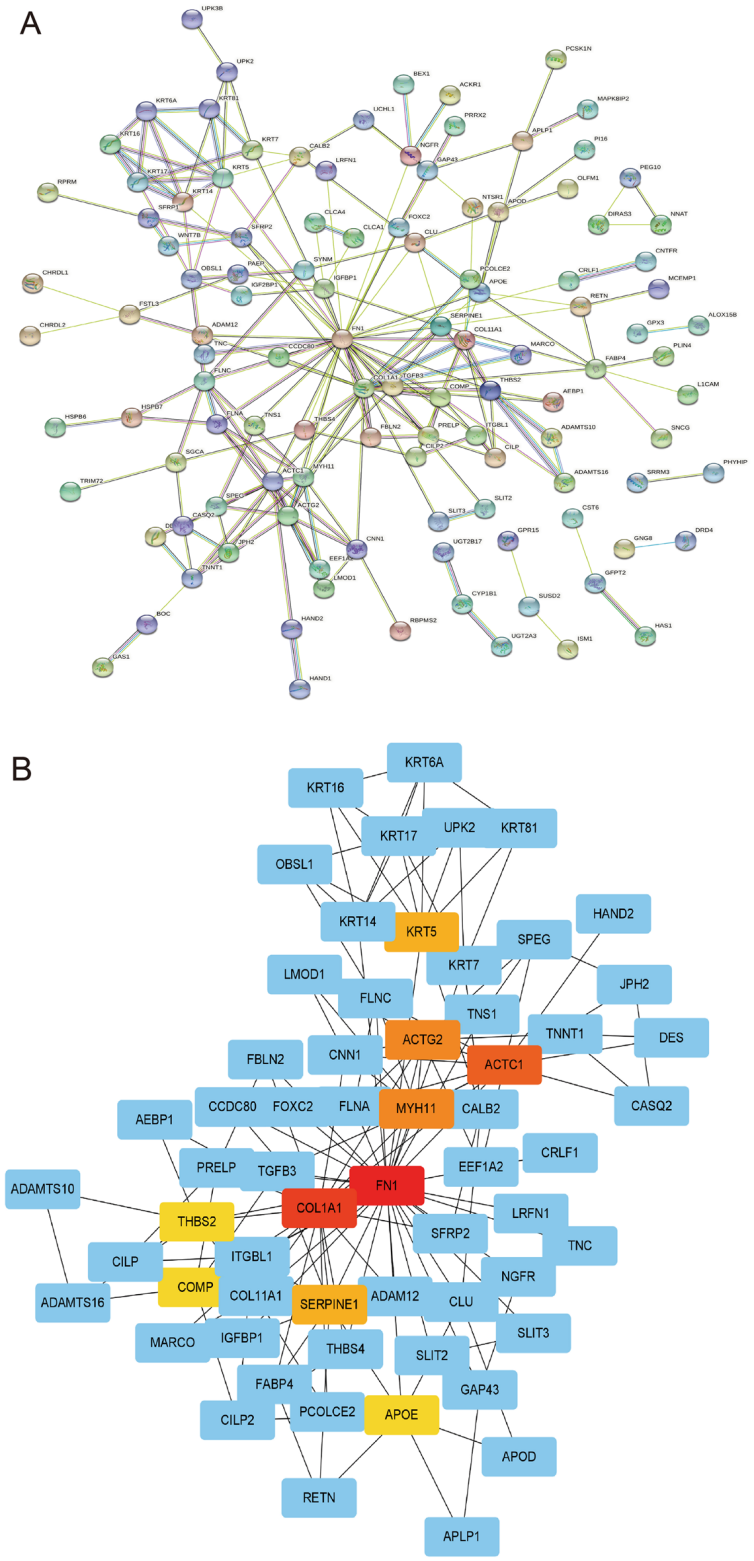
Identification of hub genes. (**A**) PPI network of differentially expressed genes between high- and low-risk groups. (**B**) Top 10 hub genes.

**Figure 9:**
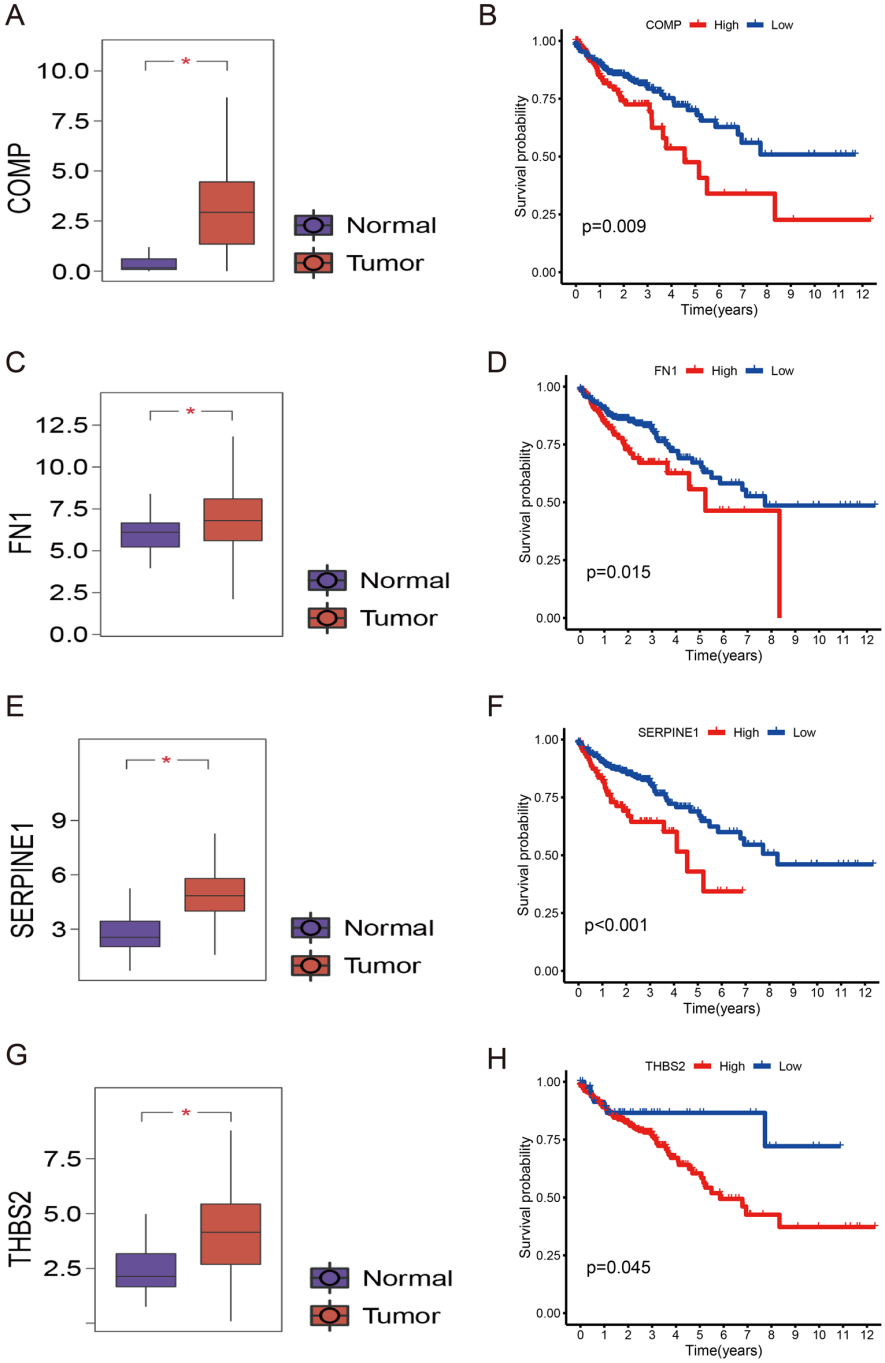
Analysis of target genes. The expression of (**A**) COMP, (**C**) FN1, (**E**) SERPINE1, and (**G**) THBS2 between normal and tumor tissues. The survival analysis of (**B**) COMP, (**D**) FN1, (**F**) SERPINE1, and (**H**) THBS2.

We constructed a PPI network using differentially expressed genes in low- and high-risk groups (Fig. 8A). We used Cytoscape software with degree algorithm to identify ten hub genes including FN1, COL1A1, ACTC1, ACTG2, MYH11, SERPINE1, KRT5, THBS2, COMP, APOE (Fig. 8B). From ten hub genes, four differentially expressed target genes (FN1, SERPINE1, THBS2, COMP) were identified using the GEPIA database, which had significant statistical differences in the survival analysis and expression analysis (p-value < 0.05). The expression levels of these four genes were higher in tumor tissues than in normal tissues (Fig. 9A,C,E,G), and the high expression of these genes had poor prognosis (Fig. 9B,D,F,H). Further analysis of these four genes was carried out next. According to the results of immune cell infiltration, the immune cells that were upregulated in the gene COMP high expression group were Macrophages M2 and resting Dendritic cells, and the downregulated immune cells were memory activated T cells CD4 and activated Dendritic cells (Supplementary Fig. 1A); the immune cells that were upregulated in the gene FN1 high expression group were Macrophages M1, and the downregulated cells were naive B cells, memory B cells, and Plasma cells (Supplementary Fig. 1B); the immune cells that were upregulated in the SERPINE1 high expression group were Macrophages M0, Macrophages M0, Macrophages M2, activated Mast cells, Eosinophils Neutrophils, memory B cells, follicular helper T cells, resting Mast cells (Supplementary Fig. 1C); THBS2 high expression group The upregulated immune cells in the expression group were Macrophages M2 and resting Dendritic cells, and the downregulated immune cells were naive B cells, memory B cells, Plasma cells, activated Dendritic cells (Supplementary Fig. 1D). Next, we explored the relationship between gene expression and clinical features of these four genes (Supplementary Figs. 2–5). Clinical correlation analysis showed that in COMP, FN1, and SERPINE1 genes, the expression levels were significantly lower in stage I patients than in stage II, III and IV patients (p-value < 0.05) (Supplementary Fig. 2E, Supplementary Fig. 3E, Supplementary Fig. 5E). Mutation analysis revealed that only FN1 has significant differences between mutant and wild types (Supplementary Fig. 6). In the target gene correlation analysis, THBS2 and other three genes (COMP, FN1, SERPINE1) were positively correlated with each other with statistically significant differences (p-value < 0.001). The relationship between COMP and FN1, SERPINE1 were also positively correlated with each other with statistically significant differences (p-value < 0.001) (Supplementary Fig. 7). Based on the above findings, it can be concluded that the high expression group had a high level of immune cell infiltration and high levels of activity, which makes it suitable for immunotherapy.

## Discussion

Colorectal cancer ranks second in mortality and third in morbidity worldwide according to statistics in 2021, which seriously affects human health^1,18^. Current treatment options for CRC are mainly surgery, chemotherapy, radiotherapy and targeted therapy, which can improve the prognosis to some extent but also have limitations, especially for patients with advanced CRC with recurrence or distant metastases. Besides, it has been reported that immunotherapy-related therapeutic strategies had the ability to overcome the limitations of classical treatment^19^. Standard conventional treatments including chemotherapy and radiotherapy have many side effects owing to their cytotoxicity and unspecificity toward any cells are growing and dividing. Immunotherapy overcomes the issue of specificity which is the major problem in chemotherapy and radiotherapy. Cancer immunotherapy targets cancer antigens of tumor cells specifically, alerts the immune systems to the presence of foreign substances and eradicates cancer through the concert of immune responses. The normal cells with no cancer antigens are not affected^20^. The principle of immunotherapy treatment is to inhibit cancer progression by activating the natural immune molecular components of the tumor microenvironment. According to reports, the cells with antitumor characteristics in the tumor microenvironment are CD8 + cytotoxic T cells, Th1 helper cells and their associated cytokines such as interferon^21^. In vivo, there is a high proportion of Th1, CD8 + T and effector memory T cells, indicating better prognosis^22^. A number of cytokines can be produced by Treg cells to enhance their ability to fight tumors^23^. In this study, the results indicated that the TIDE score in the high-risk group was higher than in the low-risk group, which could indicate that the high-risk group was more prone to immune escape, and their immunotherapy was less effective. Previous studies showed that a high TIDE score indicates a high likelihood of antitumor immune escape^24^. This is consistent with our above results. Thus, the treatment of patients with CRC may be improved by immunotherapy in the low-risk group, which is expected to have good efficacy.

Different types of cancer have different mechanisms for butyrate’s anticancer properties^25^. By activating MCT4, butyrate enhances the antitumor activity of 3-bromopyruvate in vivo and inhibits breast cancer growth^26^. The butyrate also inhibited bladder cancer cell migration by inducing autophagy and apoptosis^27^. Furthermore, butyrate inhibits cellular activity, which results in hepatocellular carcinoma and glioblastoma carcinoma that progress more slowly^28^. Butyrate has a strong relationship with CRC, and its anticancer and anti-inflammatory effects are evident in this disease^29–31^. Research on the intrinsic oncogene pathways in cancer is in full swing. However, no predictive model for butyrate metabolism-related genes has been developed in CRC so far. In the present work, the TCGA-CRC cohort was used as the training set, and the GEO-CRC cohort was used as the test set for accuracy validation. AUC value of the risk model was 0.739, 0.753 and 0.727 for 1-, 3- and 5-year respectively, exhibiting superior performance than some other signatures in predicting the prognosis of CRC. In previous studies, ferroptosis-related gene signature was constructed in CRC, and AUC was 0.64, 0.64, and 0.71 for 1-, 3- and 5-year respectively^32^. A necroptosis-related risk score model was also identified, and AUC was 0.665, 0.712, and 0.758 for the 1-, 3-, and 5-year OS^33^.

Past studies have found the intimate relationship between butyrate metabolism-related genes in the risk model and tumorigenesis and progression of CRC. As a secretory glycoprotein hormone, Stanniocalcin 2 (STC2) was involved in the progression and development of CRC through activating the Wnt/β-catenin signaling pathway and promoting CRC cell proliferation and migration^34^. Previous study have demonstrated that RNA-binding protein CELF1 targeted ETS2 in colorectal cancer, contributing to tumor cell migration, invasion and promotion of chemoresistance^35^. Insulin-like growth factor 2 RNA binding protein 1 (IGF2BP1/IMP1) has the ability to shape extracellular vesicles cargo in human CRC, and higher expression level of IGF2BP1 is correlated with poor clinical outcome^36^. FABP4 expression was associated with E-cadherin and Snail expression in CRC tissue, indicating that FABP4 may promote CRC development related to epithelial-mesenchymal transition (EMT)^37^. TIMP1 was found to be overexpressed in colon tissue and lymph node metastasis specimens, and suppression of TIMP1 expression inhibited proliferation, and metastasis but promote apoptosis through inducing FAK-PI3K/AKT and MAPK pathway^38^.

This new risk prediction model can aid in predicting the treatment outcome for patients and analyze the relationship between target genes and immune cell infiltration, clinical characteristics. In immune cell infiltration analysis, the low-risk group was enriched in Plasma cells, CD4 memory resting T cells, resting Dendritic cells, activated Dendritic cells, and Eosinophils. With their potent antigen presenting ability, dendritic cells have long been considered a key component of antitumor immunity^39^. Activated dendritic cells are key to the development of long-term and effective anticancer immunity^40^. It is well documented that eosinophils infiltrate tumors, and in most cases, this translates into a better prognosis^41^. The high-risk group is active in HLA, Type_II_IFN_Reponse and there is a study that Type_II_IFN_Reponse is essential for immune editing of tumors^42^, which indicates that the high-risk group is suitable for immunotherapy. In conclusion, targeting different targets, immunotherapy may be beneficial for both high-risk and low-risk CRC patients.

Relying on the differentially expressed genes between high and low-risk groups, we cleverly mapped the PPI network, from which we filtered the top 10 core genes of the network. We finally obtained four target genes (FN1, SERPINE1, THBS2, COMP), which were significantly different in survival analysis and expression analysis (p-value < 0.05). It is thought that these four genes are high-risk genes since they are highly expressed in tumors, and a high level of expression of these genes is associated with poor prognoses. FN1 can promote embryogenesis and host defense, as well as inhibit apoptosis while promoting the growth of CRCs in combination with ITGA5^43^. CRC aggressiveness is triggered by SERPINE1 activation by ARNTL2 and circadian rhythm variations in circulating PAI-1 levels^44^. CRC cells migrate and invade more rapidly when THBS2 regulates the Wnt/β-linked protein signaling pathway^45^. It has been reported that COMP promotes cell proliferation during the early stages of colon cancer tumorigenesis^46^. According to these studies, the results are generally consistent with ours, confirming the accuracy of the model and its scientific validity. Moreover, the ability of these four genes to act on various immune cells suggests that immunotherapy may improve survival rates in patients with poor prognoses. However, further investigation of these results is necessary.

To conclude, the butyrate prognostic model is capable of evaluating butyrate metabolism patterns comprehensively. The risk score can be used to predict chemotherapy sensitivity, as well as the prognosis of CRC patients. An effective clinical follow-up strategy can be achieved through an understanding of the risk score and the clinical stage. The prognostic model of our study will help to promote the development of new diagnostic ideas, and facilitate the search for new therapeutic targets and prognostic molecular markers in the future.

## Conclusion

In summary, a novel risk model for butyrate metabolism was developed based on data from the TCGA and GEO databases. The butyrate metabolism-related four target genes were closely related to the clinical stage and prognosis of patients with CRC and showed specificity in immune cell infiltration. It may be possible to use these four genes as biomarkers for individualized treatment of patients with CRC to improve their prognoses.

## Supplementary Information

Supplementary Legends.

Supplementary Figure 1.

Supplementary Figure 2.

Supplementary Figure 3.

Supplementary Figure 4.

Supplementary Figure 5.

Supplementary Figure 6.

Supplementary Figure 7.

Supplementary Table 1.

## Data Availability

This study relied on publicly available data. The Cancer Genome Atlas (TCGA) and Gene Expression Omnibus (GEO) databases contain this information.

## References

[1] Sung H, Ferlay J, Siegel RL, Laversanne M, Soerjomataram I, Jemal A, Bray F (2021). Global Cancer Statistics 2020: GLOBOCAN estimates of incidence and mortality worldwide for 36 cancers in 185 countries. CA Cancer J. Clin..

[2] Dollinger MM, Behl S, Fleig WE (2018). Early detection of colorectal cancer: A multi-center pre-clinical case cohort study for validation of a combined DNA stool test. Clin. Lab..

[3] Hollandsworth HM, Stringfield S, Klepper K, Zhao B, Abbadessa B, Lopez NE, Parry L, Ramamoorthy S, Eisenstein S (2020). Multiquadrant surgery in the robotic era: A technical description and outcomes for da Vinci Xi robotic subtotal colectomy and total proctocolectomy. Surg. Endosc..

[4] Gustavsson B, Carlsson G, Machover D, Petrelli N, Roth A, Schmoll HJ, Tveit KM, Gibson F (2015). A review of the evolution of systemic chemotherapy in the management of colorectal cancer. Clin. Colorectal Cancer.

[5] Sasaki N, Clevers H (2018). Studying cellular heterogeneity and drug sensitivity in colorectal cancer using organoid technology. Curr. Opin. Genet. Dev..

[6] Sagaert X, Vanstapel A, Verbeek S (2018). Tumor heterogeneity in colorectal cancer: What do we know so far?. Pathobiology.

[7] Tang J, Yan T, Bao Y, Shen C, Yu C, Zhu X, Tian X, Guo F, Liang Q, Liu Q (2019). LncRNA GLCC1 promotes colorectal carcinogenesis and glucose metabolism by stabilizing c-Myc. Nat. Commun..

[8] Arnold M, Sierra MS, Laversanne M, Soerjomataram I, Jemal A, Bray F (2017). Global patterns and trends in colorectal cancer incidence and mortality. Gut.

[9] Astbury SM, Corfe BM (2012). Uptake and metabolism of the short-chain fatty acid butyrate, a critical review of the literature. Curr. Drug Metab..

[10] Velázquez OC, Lederer HM, Rombeau JL, Kritchevsky D, Bonfield C (1997). Butyrate and the colonocyte. Dietary Fiber in Health and Disease.

[11] Shen ZH, Zhu CX, Quan YS, Yang ZY, Wu S, Luo WW, Tan B, Wang XY (2018). Relationship between intestinal microbiota and ulcerative colitis: Mechanisms and clinical application of probiotics and fecal microbiota transplantation. World J. Gastroenterol..

[12] Li Q, Cao L, Tian Y, Zhang P, Ding C, Lu W, Jia C, Shao C, Liu W, Wang D (2018). Butyrate suppresses the proliferation of colorectal cancer cells via targeting pyruvate kinase M2 and metabolic reprogramming. Mol. Cell. Proteomics.

[13] Thangaraju M, Cresci GA, Liu K, Ananth S, Gnanaprakasam JP, Browning DD, Mellinger JD, Smith SB, Digby GJ, Lambert NA (2009). GPR109A is a G-protein-coupled receptor for the bacterial fermentation product butyrate and functions as a tumor suppressor in colon. Cancer Res..

[14] Tabe Y, Konopleva M, Andreeff M (2020). Fatty acid metabolism, bone marrow adipocytes, and AML. Front. Oncol..

[15] Liberzon A, Birger C, Thorvaldsdottir H, Ghandi M, Mesirov JP, Tamayo P (2015). The molecular signatures database (MSigDB) hallmark gene set collection. Cell Syst..

[16] Kanehisa M, Goto S (2000). KEGG: Kyoto encyclopedia of genes and genomes. Nucleic Acids Res..

[17] Kanehisa M, Sato Y, Kawashima M, Furumichi M, Tanabe M (2016). KEGG as a reference resource for gene and protein annotation. Nucleic Acids Res..

[18] Li N, Lu B, Luo C, Cai J, Lu M, Zhang Y, Chen H, Dai M (2021). Incidence, mortality, survival, risk factor and screening of colorectal cancer: A comparison among China, Europe, and Northern America. Cancer Lett..

[19] Signorini L, Delbue S, Ferrante P, Bregni M (2016). Review on the immunotherapy strategies against metastatic colorectal carcinoma. Immunotherapy.

[20] Johdi NA, Sukor NF (2020). Colorectal cancer immunotherapy: Options and strategies. Front. Immunol..

[21] Taube JM, Galon J, Sholl LM, Rodig SJ, Cottrell TR, Giraldo NA, Baras AS, Patel SS, Anders RA, Rimm DL (2018). Implications of the tumor immune microenvironment for staging and therapeutics. Mod. Pathol..

[22] Pages F, Galon J, Dieu-Nosjean MC, Tartour E, Sautes-Fridman C, Fridman WH (2010). Immune infiltration in human tumors: A prognostic factor that should not be ignored. Oncogene.

[23] Ferreira C, Barros L, Baptista M, Blankenhaus B, Barros A, Figueiredo-Campos P, Konjar S, Laine A, Kamenjarin N, Stojanovic A (2020). Type 1 Treg cells promote the generation of CD8(+) tissue-resident memory T cells. Nat. Immunol..

[24] Fu J, Li K, Zhang W, Wan C, Zhang J, Jiang P, Liu XS (2020). Large-scale public data reuse to model immunotherapy response and resistance. Genome Med..

[25] O'Keefe SJ (2016). Diet, microorganisms and their metabolites, and colon cancer. Nat. Rev. Gastroenterol. Hepatol..

[26] Queiros O, Preto A, Pacheco A, Pinheiro C, Azevedo-Silva J, Moreira R, Pedro M, Ko YH, Pedersen PL, Baltazar F (2012). Butyrate activates the monocarboxylate transporter MCT4 expression in breast cancer cells and enhances the antitumor activity of 3-bromopyruvate. J. Bioenerg. Biomembr..

[27] Wang F, Wu H, Fan M, Yu R, Zhang Y, Liu J, Zhou X, Cai Y, Huang S, Hu Z (2020). Sodium butyrate inhibits migration and induces AMPK-mTOR pathway-dependent autophagy and ROS-mediated apoptosis via the miR-139-5p/Bmi-1 axis in human bladder cancer cells. FASEB J..

[28] Joachimiak R, Kaznica A, Drewa T (2007). Influence of sodium butyrate on hepatocellular carcinoma (hepG2) and glioblastoma (C6) cell lines in vitro. Acta Pol. Pharm..

[29] Hassig CA, Tong JK, Schreiber SL (1997). Fiber-derived butyrate and the prevention of colon cancer. Chem. Biol..

[30] Velazquez OC, Rombeau JL (1997). Butyrate: Potential role in colon cancer prevention and treatment. Adv. Exp. Med. Biol..

[31] Ooi CC, Good NM, Williams DB, Lewanowitsch T, Cosgrove LJ, Lockett TJ, Head RJ (2010). Structure-activity relationship of butyrate analogues on apoptosis, proliferation and histone deacetylase activity in HCT-116 human colorectal cancer cells. Clin. Exp. Pharmacol. Physiol..

[32] Hu D, Zhou Z, Wang J, Zhu K (2022). Screening of ferroptosis-related genes with prognostic effect in colorectal cancer by bioinformatic analysis. Front. Mol. Biosci..

[33] Yang W, Lu S, Peng L, Zhang Z, Zhang Y, Guo D, Ma F, Hua Y, Chen X (2022). Integrated analysis of necroptosis-related genes for evaluating immune infiltration and colon cancer prognosis. Front. Immunol..

[34] Li Q, Zhou X, Fang Z, Pan Z (2019). Effect of STC2 gene silencing on colorectal cancer cells. Mol. Med. Rep..

[35] Wang H, Huang R, Guo W, Qin X, Yang Z, Yuan Z, Wei Y, Mo C, Zeng Z, Luo J (2020). RNA-binding protein CELF1 enhances cell migration, invasion, and chemoresistance by targeting ETS2 in colorectal cancer. Clin. Sci. (Lond.).

[36] Kuhn M, Zhang Y, Favate J, Morita M, Blucher A, Das S, Liang S, Preet R, Parham LR, Williams KN (2022). IMP1/IGF2BP1 in human colorectal cancer extracellular vesicles. Am. J. Physiol. Gastrointest. Liver Physiol..

[37] Zhang Y, Zhang W, Xia M, Xie Z, An F, Zhan Q, Tian W, Zhu T (2021). High expression of FABP4 in colorectal cancer and its clinical significance. J. Zhejiang Univ. Sci. B.

[38] Song G, Xu S, Zhang H, Wang Y, Xiao C, Jiang T, Wu L, Zhang T, Sun X, Zhong L (2016). TIMP1 is a prognostic marker for the progression and metastasis of colon cancer through FAK-PI3K/AKT and MAPK pathway. J. Exp. Clin. Cancer Res..

[39] Veglia F, Gabrilovich DI (2017). Dendritic cells in cancer: The role revisited. Curr. Opin. Immunol..

[40] Gilardini Montani MS, D'Eliseo D, Cirone M, Di Renzo L, Faggioni A, Santoni A, Velotti F (2015). Capsaicin-mediated apoptosis of human bladder cancer cells activates dendritic cells via CD91. Nutrition.

[41] Davis BP, Rothenberg ME (2014). Eosinophils and cancer. Cancer Immunol. Res..

[42] Di Franco S, Turdo A, Todaro M, Stassi G (2017). Role of type I and II interferons in colorectal cancer and melanoma. Front. Immunol..

[43] Cai X, Liu C, Zhang TN, Zhu YW, Dong X, Xue P (2018). Down-regulation of FN1 inhibits colorectal carcinogenesis by suppressing proliferation, migration, and invasion. J. Cell. Biochem..

[44] Mazzoccoli G, Pazienza V, Panza A, Valvano MR, Benegiamo G, Vinciguerra M, Andriulli A, Piepoli A (2012). ARNTL2 and SERPINE1: Potential biomarkers for tumor aggressiveness in colorectal cancer. J. Cancer Res. Clin. Oncol..

[45] Qu HL, Hasen GW, Hou YY, Zhang CX (2022). THBS2 promotes cell migration and invasion in colorectal cancer via modulating Wnt/beta-catenin signaling pathway. Kaohsiung J. Med. Sci..

[46] Nfonsam VN, Jecius HC, Janda J, Omesiete PN, Elquza E, Scott AJ, Nfonsam LE, Jandova J (2020). Cartilage oligomeric matrix protein (COMP) promotes cell proliferation in early-onset colon cancer tumorigenesis. Surg. Endosc..

