# Supplementary material for: Characterization of butyrate-metabolism in colorectal cancer to guide clinical treatment
Source: Sci Rep. 2023 Mar 29;13:5106. doi: 10.1038/s41598-023-32457-z (PMC10060236; doi:10.1038/s41598-023-32457-z)
Supplement: Supplementary file 1 — Supplementary Legends. [file 41598_2023_32457_MOESM1_ESM.docx]

**Supplementary Figure 1**. Immune cell infiltration analysis of (A) COMP, (B) FN1, (C) SERPINE1, and (D) THBS2.

**Supplementary Figure 2**. Clinical correlation analysis of COMP, including (A)age, (B) gender, (C) M stage, (D) N stage, (E)stage, (F) T stage.

**Supplementary Figure 3**. Clinical correlation analysis of FN1, including (A)age, (B) gender, (C) M stage, (D) N stage, (E)stage, (F) T stage.

**Supplementary Figure 4**. Clinical correlation analysis of THBS2, including (A)age, (B) gender, (C) M stage, (D) N stage, (E)stage, (F) T stage.

**Supplementary Figure 5**. Clinical correlation analysis of SERPINE1, including (A)age, (B) gender, (C) M stage, (D) N stage, (E)stage, (F) T stage.

**Supplementary Figure 6**. Correlation analysis between the type of gene mutation (mutant, wild type) and risk score, including (A) COMP, (B)FN1, (C) SERPINE1, and (D) THBS2.

**Supplementary Figure 7**. Correlation analysis between target genes. (A) Correlation analysis between COMP and SERPINE1. (B) Correlation analysis between FN1 and COMP. (D) Correlation analysis between THBS2 and FN1. (C) Correlation analysis between THBS2 and COMP. (E) Correlation analysis between THBS2 and SERPINE1.

**Supplementary Table 1.** Drug sensitivity associated with high- and low-risk patients in CRC.
