# Supplementary figures and images for: Characterization of butyrate-metabolism in colorectal cancer to guide clinical treatment
Source: Sci Rep. 2023 Mar 29;13:5106. doi: 10.1038/s41598-023-32457-z (PMC10060236; doi:10.1038/s41598-023-32457-z)

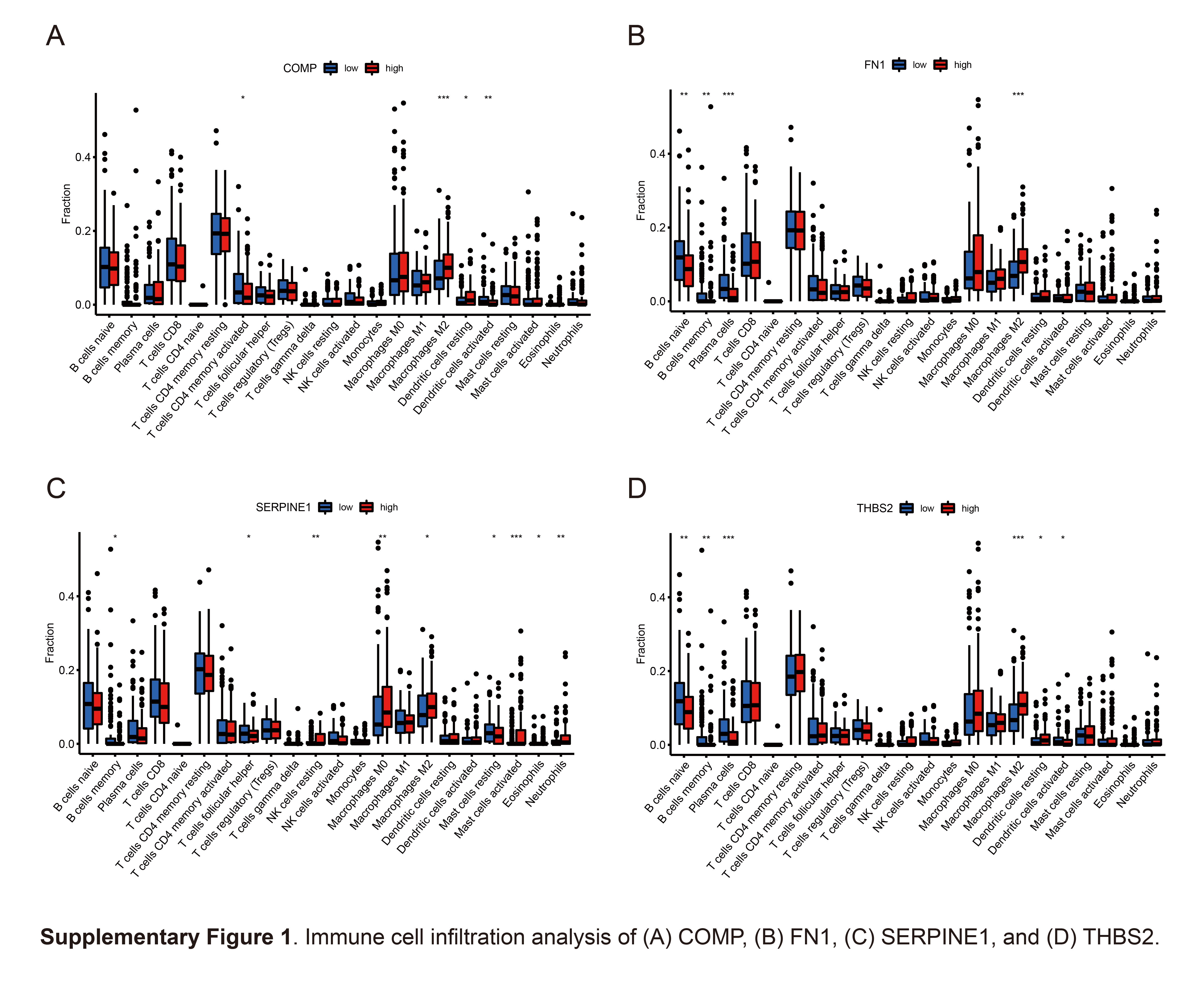

Supplement: Supplementary file 2 — Supplementary Figure 1. [file 41598_2023_32457_MOESM2_ESM.jpg]

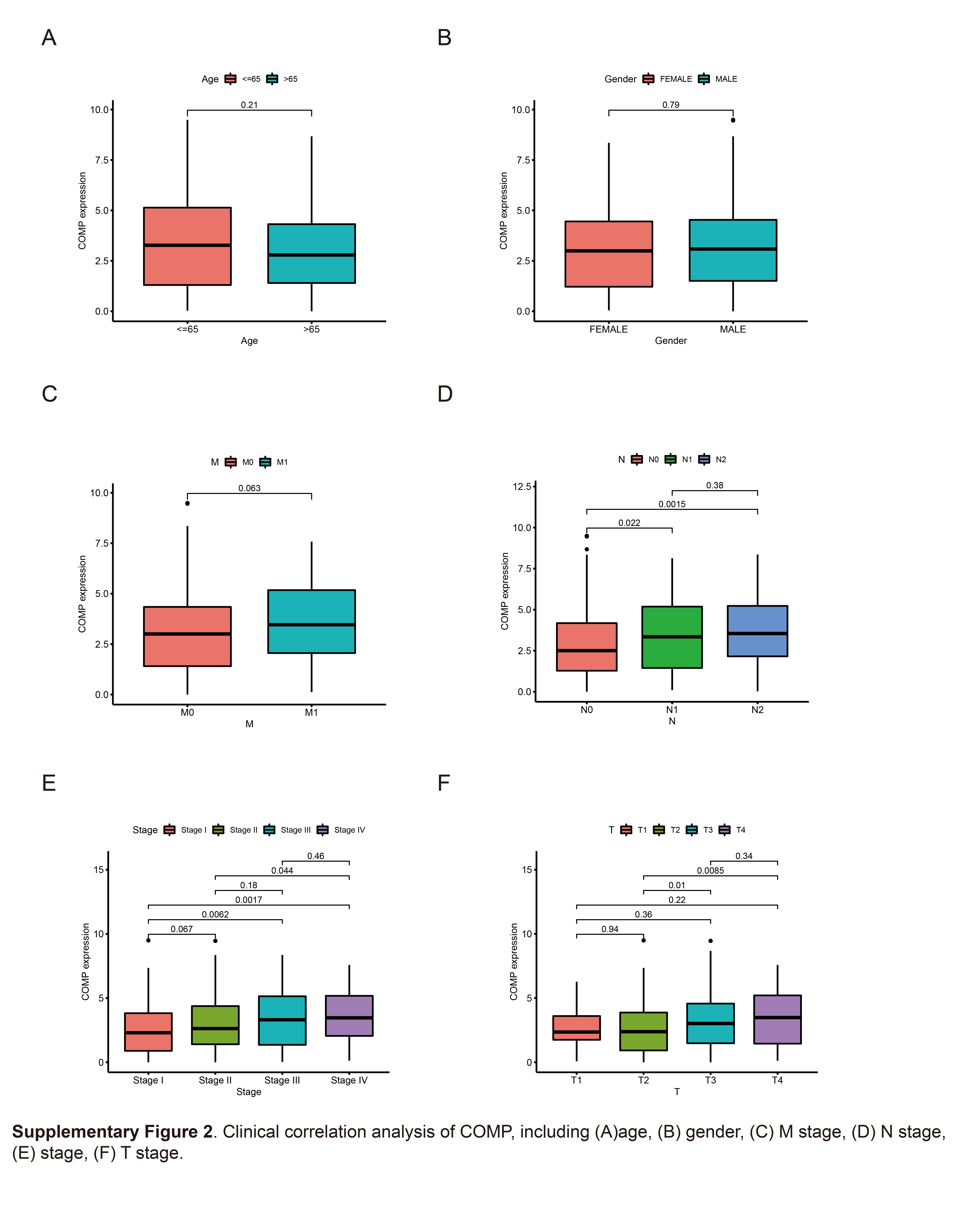

Supplement: Supplementary file 3 — Supplementary Figure 2. [file 41598_2023_32457_MOESM3_ESM.jpg]

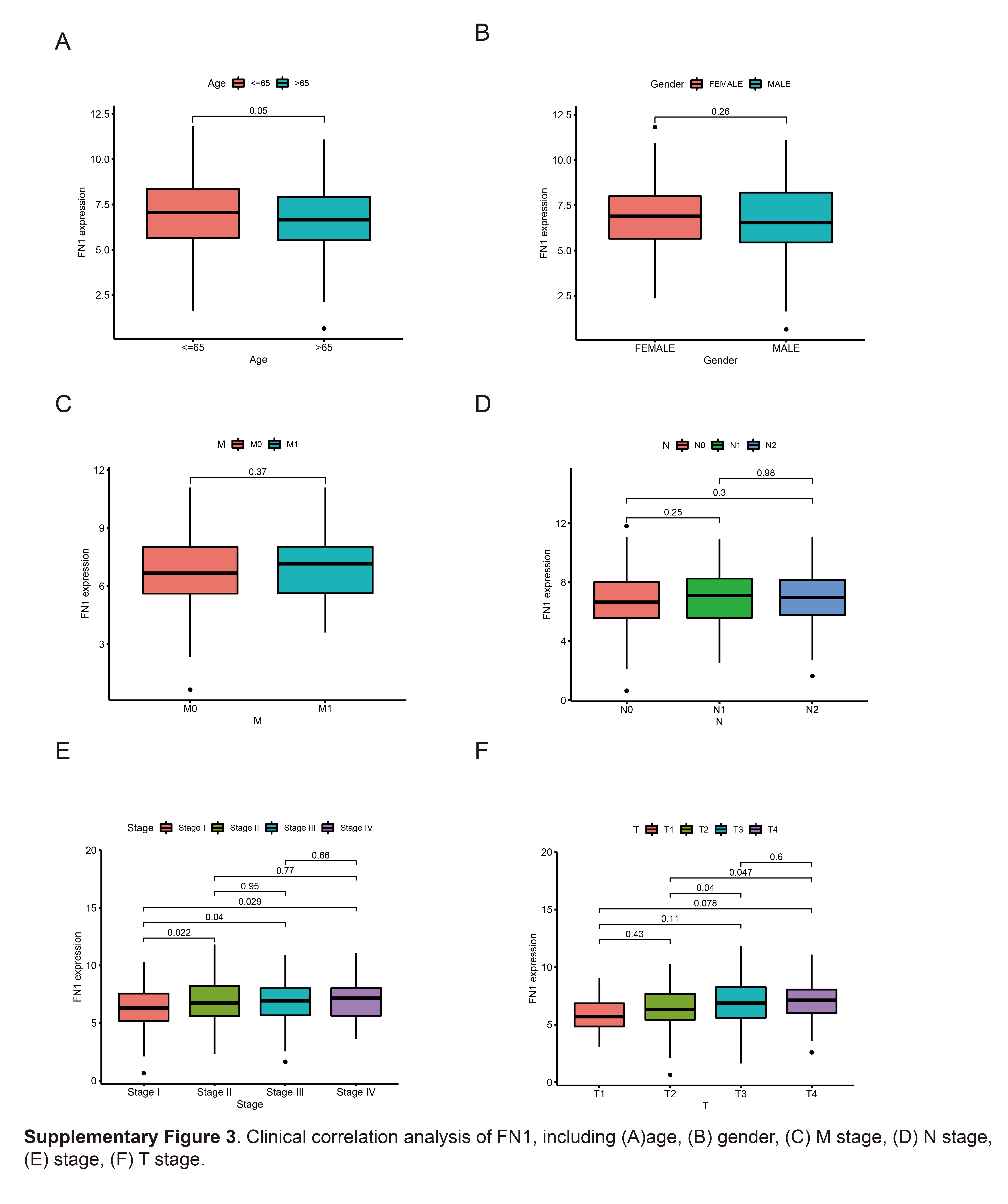

Supplement: Supplementary file 4 — Supplementary Figure 3. [file 41598_2023_32457_MOESM4_ESM.jpg]

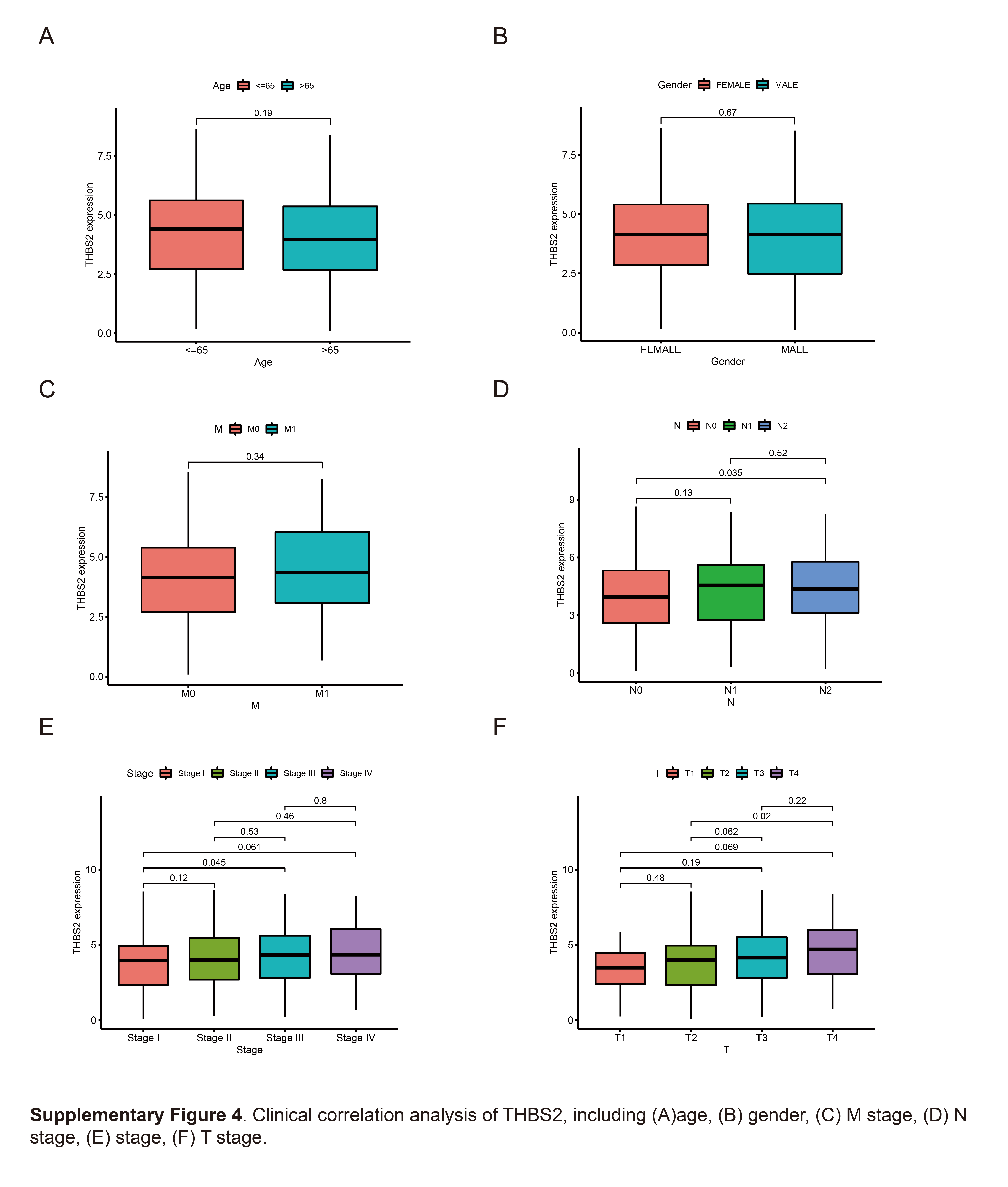

Supplement: Supplementary file 5 — Supplementary Figure 4. [file 41598_2023_32457_MOESM5_ESM.jpg]

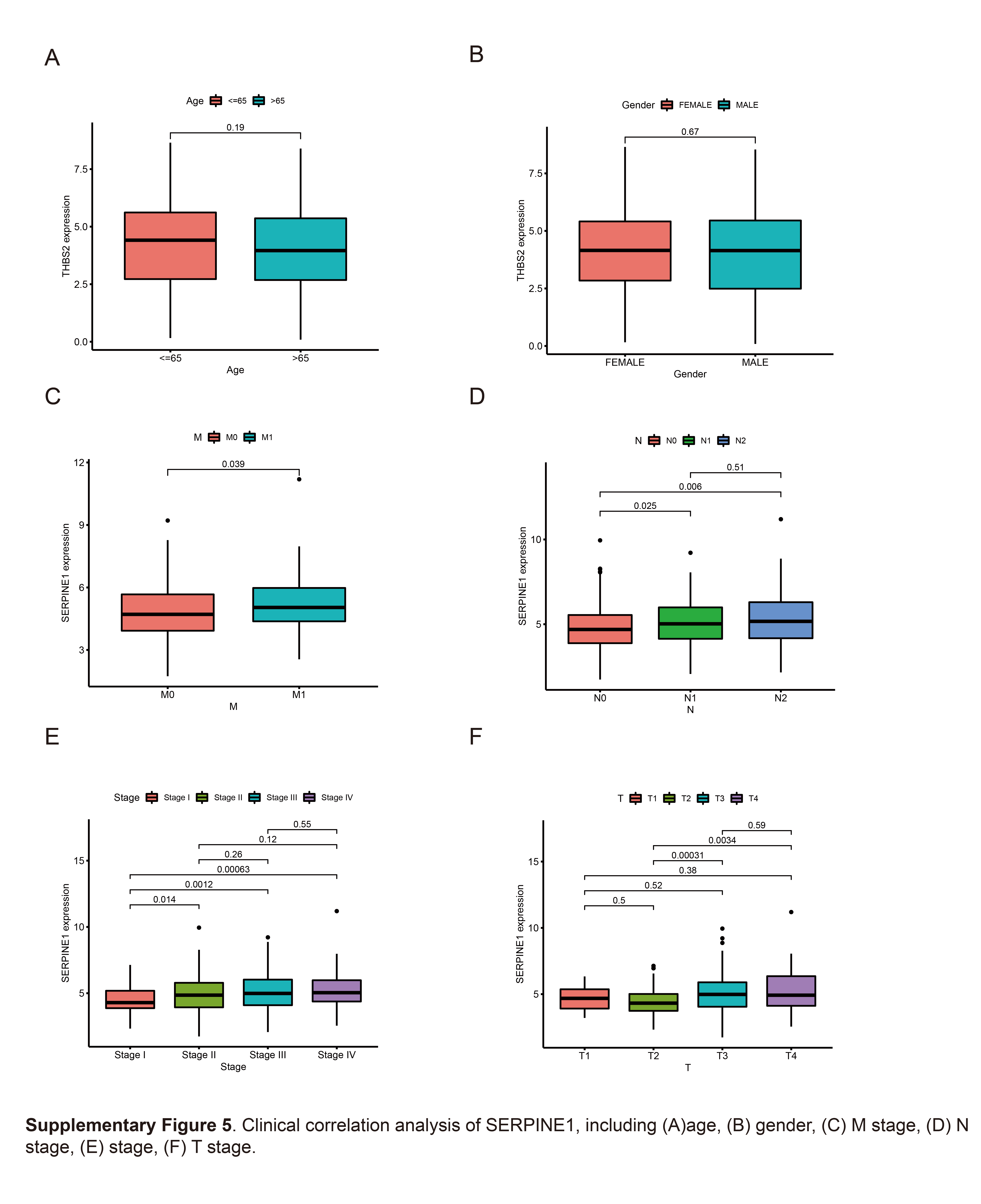

Supplement: Supplementary file 6 — Supplementary Figure 5. [file 41598_2023_32457_MOESM6_ESM.jpg]

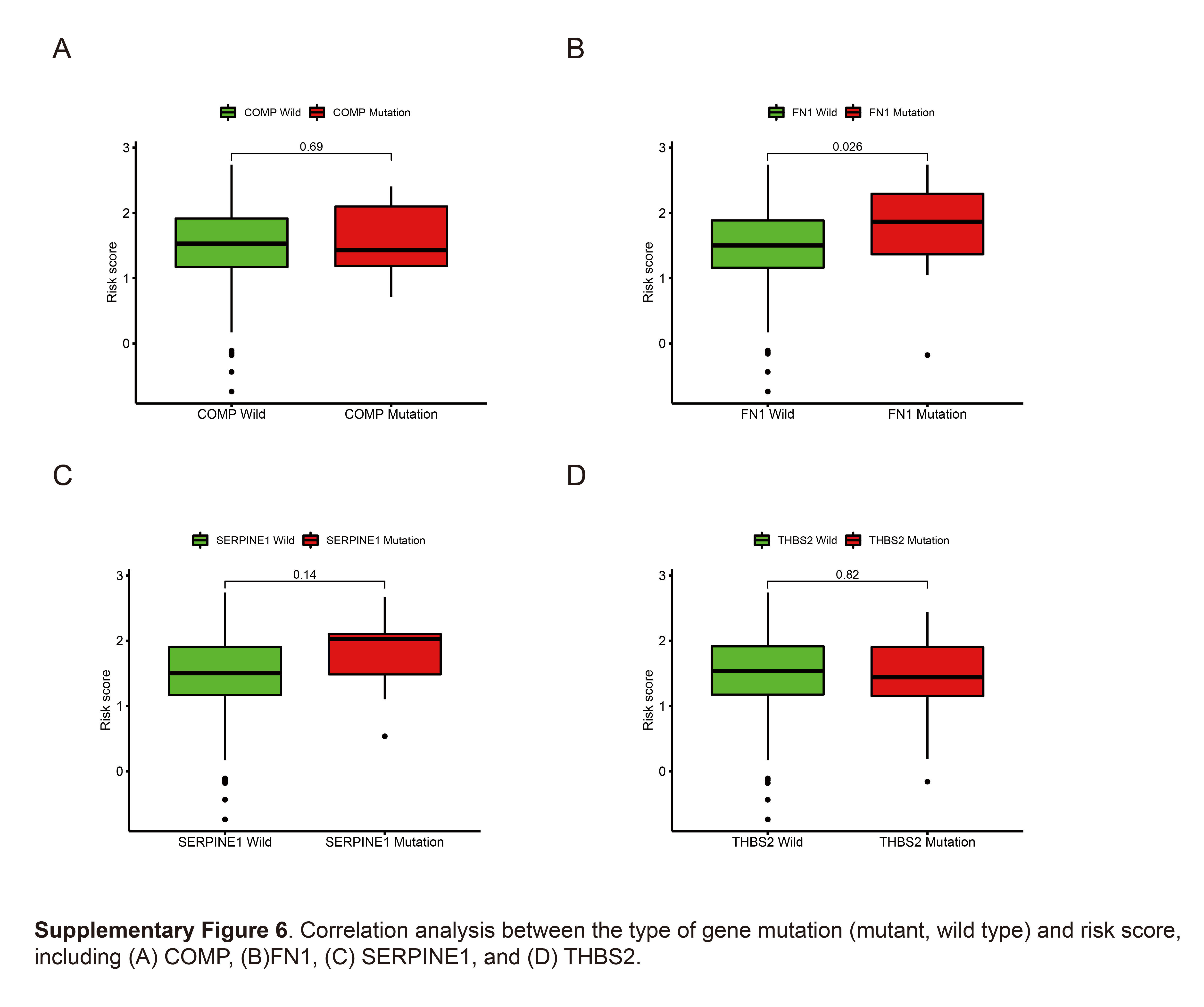

Supplement: Supplementary file 7 — Supplementary Figure 6. [file 41598_2023_32457_MOESM7_ESM.jpg]

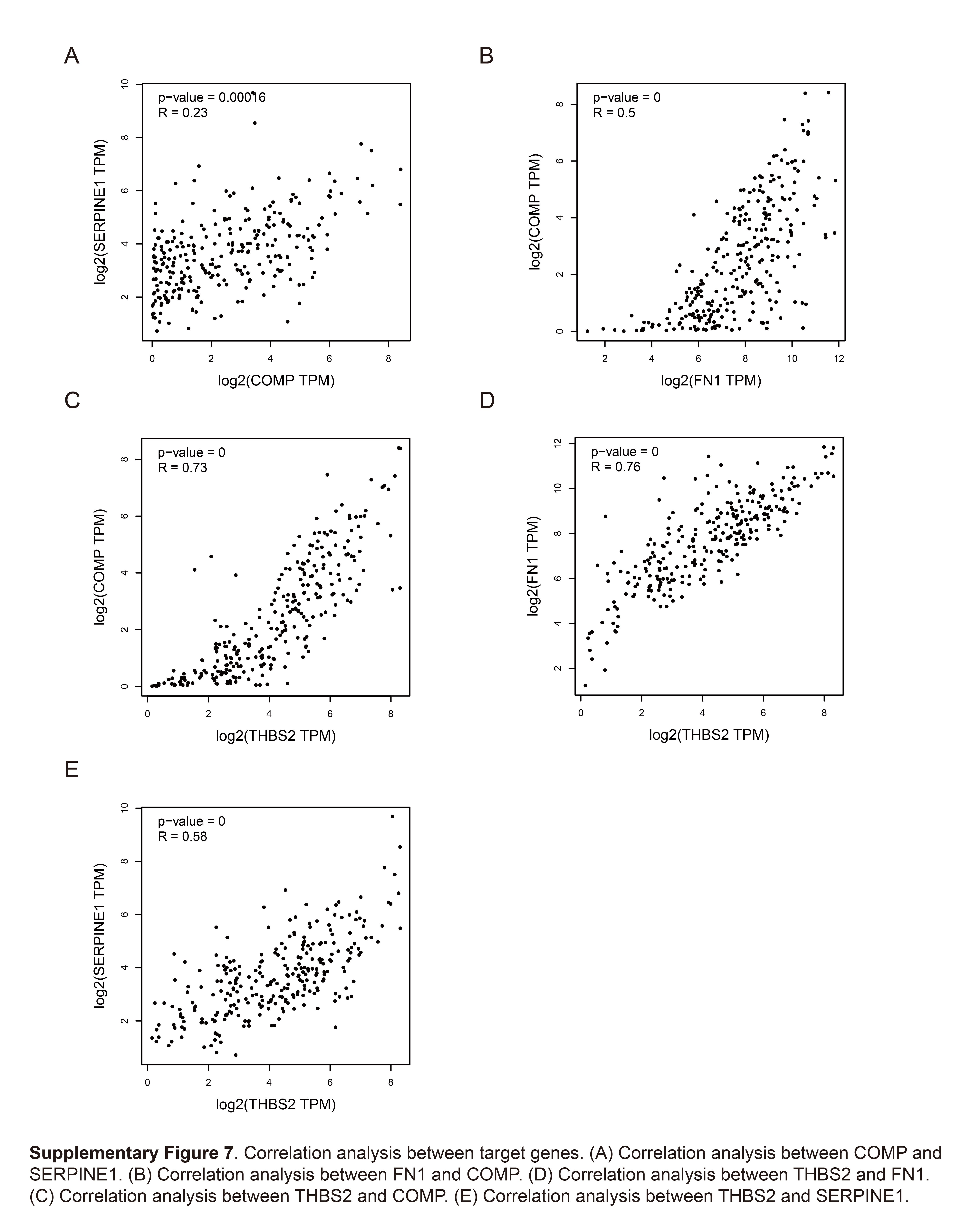

Supplement: Supplementary file 8 — Supplementary Figure 7. [file 41598_2023_32457_MOESM8_ESM.jpg]
